# The H-HOPE behavioral intervention plus Kangaroo Mother Care increases mother-preterm infant responsivity in Malawi: a prospective cohort comparison

**DOI:** 10.1186/s12887-023-04015-z

**Published:** 2023-04-21

**Authors:** Esnath M. Kapito, Ellen M. Chirwa, Elizabeth Chodzaza, Kathleen F. Norr, Crystal Patil, Alfred O. Maluwa, Rosemary White-Traut

**Affiliations:** 1 School of Maternal, Neonatal and Reproductive Health Studies, Kamuzu University of Health Sciences, Private Bag 360, Blantyre, Malawi; 2 Children’s Hospital of Wisconsin, Children’s Research Institute, 9000 Winsconsin Avenue, Milwaukee, Winsconsin 53226 USA; 3 College of Nursing, University of Illinois Chicago, 845 S. Damen Avenue (M/C 806), Chicago, IL 60612 USA; 4 Malawi University of Science and Technology, P. O Box 5196, Limbe, Malawi

**Keywords:** Mother-infant responsivity, Preterm birth, Kangaroo Mother Care (KMC), Early behavioral intervention, H-HOPE, Malawi

## Abstract

**Background:**

Early behavioral intervention to promote development is recommended as the standard of care for preterm infants, yet is not provided in Malawi. One such intervention is H-HOPE (Hospital to Home: Optimizing the Premature Infant’s Environment). In US studies, H-HOPE increased mother-preterm infant responsivity at 6-weeks corrected age (CA). Kangaroo Mother Care (KMC) improves infant survival and is the standard of care for preterm infants in Malawi. This is the first study to examine whether H-HOPE is feasible and promotes mother-preterm infant responsivity in Malawi, and the first to examine the impact of H-HOPE when KMC is the standard of care.

**Method:**

This pilot was conducted in a KMC unit using a prospective cohort comparison design. Because the unit is an open room without privacy, random assignment would have led to contamination of the control cohort. H-HOPE includes participatory guidance for mothers and Massage + , a 15 min multisensory session provided by mothers twice daily. H-HOPE began when infants were clinically stable and at least 32 weeks postmenstrual age. Mothers participated if they were physically stable and willing to return for follow-up. Mother-preterm infant dyads were video-recorded during a play session at 6-weeks CA. Responsivity was measured using the Dyadic Mutuality Code (DMC).

**Results:**

The final sample included 60 H-HOPE + KMC and 59 KMC only mother-preterm infant dyads. Controlling for significant maternal and infant characteristics, the H-HOPE + KMC dyads were over 11 times more likely to have higher responsivity than those in the KMC only dyads (AOR = 11.51, CI = 4.56, 29.04). The only other factor related to higher responsivity was vaginal vs. Caesarian delivery (AOR = 5.44, CI = .096, 30.96).

**Conclusion:**

This study demonstrated that H-HOPE can be provided in Malawi. Mother-infant dyads receiving both H-HOPE and KMC had higher responsivity at 6-weeks CA than those receiving KMC only. H-HOPE was taught by nurses in this study, however the nursing shortage in Malawi makes H-HOPE delivery by nurses challenging. Training patient attendants in the KMC unit is a cost-effective alternative. H-HOPE as the standard of care offers benefits to preterm infants and mothers that KMC alone does not provide.

## Background

Preterm birth, defined as birth prior to 37 weeks gestational age, remains a major global public health concern with about 15 million preterm births occurring annually [1]. Complications of prematurity include difficulty regulating body temperature, breathing, and feeding and accounts for 35% of neonatal deaths [2]. Even when preterm neonates have no major neurosensory and motor impairments, they are at higher risk for developmental, behavioral, and socio-emotional problems [3–8]. Prematurity also negatively affects parents; they experience increased stress, anxiety, and depressive symptoms, have lower parenting confidence, and less optimal parent-infant interaction. Collectively, this adversely impacts parent-infant interaction and infant growth and development [9–12].

Malawi carries a high burden of preterm birth at 18%, well above the global rate of 11% [13–16]. Complications of prematurity in Malawi account for 36% of neonatal deaths [17] and contribute substantially to high neonatal and infant mortality rates (26 and 56 per 1,000, respectively) [18]. Malawian mothers with premature babies express fears about survival and long-term health and development of their small and fragile infants [19–21]. Additionally, prematurity is stigmatized because this is interpreted as a punishment from supernatural powers or as a consequence of having a disease, such as HIV [22].

One well-established intervention that that has been shown to reduce preterm infant morbidities and mortality is Kangaroo Mother Care (KMC), or near-constant skin-to-skin contact between the infant and mother [23]. In KMC, the infant is held inside the mother’s clothing with access to the breast for feeding. KMC has been shown to improve infant thermoregulation, nutrition, and organized sleep and reduced risk for morbidities and mortality [24–26]. KMC is especially effective in low income countries that often lack equipment and services [24, 27]. When KMC was implemented in tertiary hospitals in Ghana, India, Malawi, Nigeria and Tanzania, the data showed that KMC was associated with significantly higher neonatal survival rates [26]. Since 2005, KMC has been the standard of care in Malawi [28]. However, mothers in Malawi practicing KMC still experience the high stress, anxiety and stigma associated with a preterm birth. KMC exacerbates this stress because health facility policies do not allow visitors on the KMC Unit. Therefore, mothers are alone with their infants while providing 24-h KMC without support [21]**.** An additional stigma is associated with KMC because in Malawi mothers are expected to carry babies on their backs and not on their chest [19]. Even though mothers are practicing KMC and in near-continuous contact with their infant, their fears and stressors lead to a reluctance to engage with the neonate [19, 20, 22, 29, 30].

Another well-established intervention that is beneficial to preterm infants is an early behavioral intervention that promotes development. Developmentally-based interventions have positive impacts on brain maturation, feeding, health and development, and parent-infant interaction [31–33]. These benefits led the WHO to recommend early developmental interventions for preterm infants [34] and in the United States (US), the Physical Environment Exploratory Group endorsed these interventions as the standard of care [35]. However, early behavioral interventions that are developmentally-based have not yet been implemented to promote premature infant development in Malawi.

The only early behavioral intervention for preterm infants with both well-established efficacy and a standardized protocol is H-HOPE (Hospital to Home: Optimizing the Preterm Infant Environment) [36]. H-HOPE has both parent- and infant-focused components. The infant-focused component was developed first and is a multisensory intervention that provides auditory, tactile, visual, and vestibular stimulation. Originally called ATVV, the infant component is now called Massage + for easier recognition by parents. Because parents of preterm infants continued to experience high stress and anxiety, H-HOPE added a component for parents called Parents + , which uses participatory guidance to help parents to read, interpret and respond to preterm infant cues and provide Massage + for their infant. In a US randomized controlled trial, the full H-HOPE intervention, including both Parents + and Massage + , yielded significant positive intervention effects including infant alertness prior to feeding, feeding readiness behavior, efficiency of sucking, feeding progression, more rapid growth and development, and lower initial hospitalization costs at discharge [37–45]. At 6-weeks CA, the H-HOPE cohort had increased mother-infant social interaction and responsivity and fewer illness visits at 6-weeks CA [37, 38, 46, 47]. Earlier studies of Massage + /ATVV alone also found improved parent-infant interaction including mother-infant mutual responsivity, maternal sensitivity toward the infant and social emotional growth fostering behaviors, infant responsivity toward the mother and infant clarity of cues [46, 48]. Increased mother-infant responsivity is also linked to infant language development [49].

Although the full H-HOPE intervention has not been tested in low- and middle-income countries (LMIC), several studies found that Massage + alone (ATVV) had positive impacts in these settings. In Colombia, first time breastfeeding mothers of full-term infants were allocated to a control or intervention group (ATVV provided for at least 2 weeks at home). In the intervention group, infant sucking ability and growth increased, maternal stress and postpartum depression decreased, and mother-infant responsivity at 6-weeks corrected age (CA) as measured by the Dyadic Mutuality Code (DMC) increased [50]. Similar results were reported from India [51]. As measured in the Infant Neurological International Battery (INFANIB) preterm infants in the ATVV arm had better motor development and tonal maturation than those who received routine care when they reached term age. However, these studies were not fully comparable to the US study as one did not use the DMC and the other did not study preterm infants.

Despite these benefits, an early behavioral intervention such as H-HOPE has never been tested for preterm infants and mothers in Malawi or any other African country. Because KMC is the standard of care for stable preterm infants in Malawi, any study would need to compare H-HOPE plus KMC with KMC only. KMC supports near constant mother-preterm infant skin-toskin contact, thermoregulation and constant access to breastfeeding, while H-HOPE fosters face-to-face positioning, mother-infant interaction and mutual engagement. Thus, the benefits of KMC and H-HOPE are complementary and preterm infants and parents should benefit from receiving both interventions. However, no prior studies have examined the impact of providing both H-HOPE and KMC, and only one study has directly compared the infant component of H-HOPE (Massage +) and KMC. That study, conducted in the US where mothers provided modified KMC with only intermittent skin-to-skin contact, found that premature infants receiving skin-to-skin contact exhibited significantly fewer social interactive behaviors than infants who received Massage + [52].

The purpose of this paper is to test the feasibility of offering H-HOPE + KMC and to compare mother-preterm infant dyads’ responsivity at 6-weeks CA for dyads receiving H-HOPE + KMC versus those receiving only KMC. As far as we know based on extensive review of published studies, this is the first study to examine the feasibility of providing H-HOPE for preterm infants in Africa, and the first study to examine the impact of providing H-HOPE along with KMC on mother-infant responsivity in any country.

## Methods

### Design

A prospective cohort design was used to compare responsivity at 6-weeks CA among dyads receiving H-HOPE and KMC and those receiving only KMC.

### Setting

The study was conducted at the KMC unit of the Neonatal Nursery Ward at Zomba Central Hospital, one of the four central hospitals in Malawi, where infants born in this hospital and those referred from other health centers in Zomba and nearby districts get care. In 2019, 2563 neonates were admitted to the neonatal nursery; once clinically stable 572 preterm infants were admitted to the KMC unit where they receive continuous skin-to-skin contact with their mothers (Zomba Central Hospital Registry data, 2019). The KMC unit is an open room with 10 beds that does not provide audio or visual privacy for mother-infant dyads. To control for infections, no outside visitors can enter the KMC unit.

An informally trained patient attendant is always present on the KMC unit. Patient attendants teach KMC, measure infant temperature and weight each day, and assist with feeding. Each day one nurse-midwife and clinical officer, a physician, complete rounds. Once discharged, mother-infant dyads within the catchment area return to the KMC unit for physical assessments and follow-up appointments. However, the dyads who are not from the catchment area of ZCH are reviewed at their nearest health center. Although H-HOPE is suitable for any caregiver, in this study we targeted the mothers because they were on the KMC unit with their infants 24 h a day until discharged.

### Estimated sample size

Our power analysis was informed by a US clinical trial that reported an effect size of 0.30 for the impact of H-HOPE on mother-infant responsivity [46]. Given the 24 h skin-to-skin care practices and cultural beliefs surrounding infant care in Malawi, we expected H-HOPE to have an even greater impact on mother-infant responsivity and selected an effect size of 0.45. We set power at 0.80 and the 2-sided confidence interval at 95%. Using the approach of Schmidt et al. [53], we calculated the minimum sample size using the formula: 2SD (Zɑ/2 + Zβ)^2^ y/d^2^), which resulted in a minimum sample size of 118 mother-preterm infant dyads, 59 mother-preterm infant dyads in each cohort (KMC only or H-HOPE + KMC) at 6-weeks CA when mother-infant responsivity is assessed. We then adjusted for a 75% return rate at 6-weeks CA [54], which would require initial enrollment of 148, or 74 per cohort.

### Inclusion criteria and recruitment

Infant inclusion criteria were: birth weight between 950–2400 g; gestational age between 29–34 weeks at birth (based on a prenatal ultrasound or the Modified Ballard Index [55] when ultrasound data were not available); and clinically stable with no severe neurological problems such as hydrocephalus or Down’s syndrome. When multiples were born, one infant was randomly selected for inclusion in the study. Mothers were eligible if they were willing to participate, physically stable, and would return to the hospital rather than their local clinic for follow-up.

Because the attrition rate for the first cohort (KMC only) was higher than our projected 25% at 6-weeks CA (37%), we increased the sample to 100 dyads per cohort to ensure that the sample was large enough to adequately compare the impact of KMC only and H-HOPE + KMC (the intervention cohort) on mother-infant responsivity. This adjustment resulted in two KMC only cohorts, one recruited before and one recruited after, the H-HOPE + KMC cohort.

**Fig. 1:**
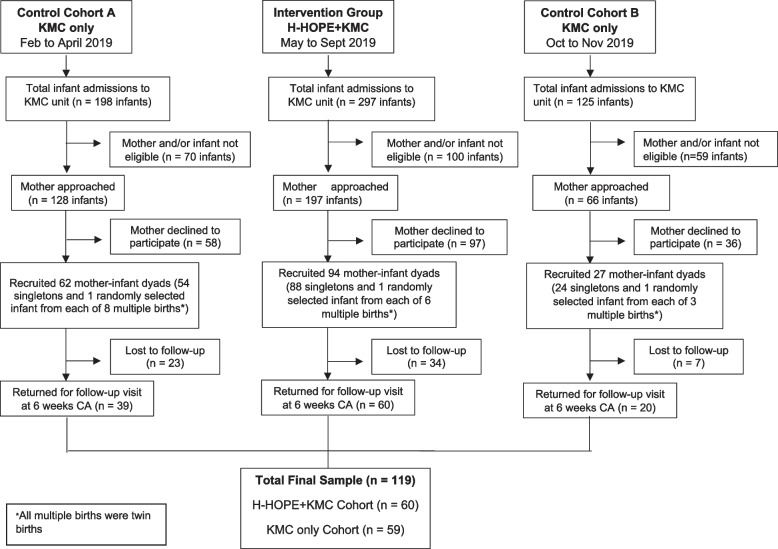
Recruitment and retention for KMC only (2 cohorts) and H-HOPE + KMC

A total of 391 infants met inclusion criteria and their mothers were approached for consent (Fig. 1). One hundred ninety-one mothers (48.8%) declined to participate for various reasons. A total of 183 mother-preterm infant dyads were enrolled with 89 in the KMC only cohort (control) and 94 in the H-HOPE + KMC cohort (intervention). At 6-weeks CA, 65% were retained (66.3% from the control cohort and 64.9% in the H-HOPE + KMC cohort). There were no significant differences in baseline maternal and infant characteristics comparing those who were retained and those lost to follow-up. The final sample at 6-weeks CA included 119 mother-preterm infant dyads for analysis (KMC only, *n* = 59; H-HOPE + KMC, *n* = 60).

### Study conditions

#### KMC (Standard of Care)

The Zomba Central Hospital practices near constant KMC. Mothers of stable preterm infants weighing at least 1500 g are admitted to the KMC ward where patient attendants supervise mothers. Mothers are encouraged to always put their infants in the KMC position except when they are going to the bathroom or outside to receive visitors. They are taught how to put the baby in the KMC position and advised to practice near constant skin-to-skin contact 24 h per day. Mothers are advised to sleep in a half-sitting position to maintain the baby in a vertical position. Immediate and exclusive breastfeeding is advised for all infants receiving KMC who are able to suck. Infants who are unable to suck effectively are frequently fed with expressed breast milk by cup or nasogastric tube, depending on the presence of the swallowing reflex.

#### H-HOPE + KMC

Mothers in the H-HOPE + KMC group received the H-HOPE intervention as well as KMC. H-HOPE consists of a parent component (Parents +) and an infant component (Massage +). The Parents + component has four sessions, which in this study were provided by the research assistant. In the first session, mothers learn how to read, interpret, and respond to their infant’s cues including behavioral states, engagement and disengagement, orally directed behaviors, and hunger and satiation and how to provide Massage + . Mothers then provided Massage + for 15 min prior to a feeding twice a day. The second session occurred near discharge and reviewed cues and Massage + and discussed going home, soothing a fussy baby and signs and symptoms of infant illness. Mothers were given a log and asked to continue providing Massage + twice daily until the infant reached 6-weeks CA. Sessions 3 and 4 occurred during follow-up visits at 1- 2 weeks after discharge and 6-weeks CA. Content included review of previous learning including Massage + and log review, maternal and infant symptoms that require attention and when to access care. Mothers also were asked about support at home, infant feeding and any new concerns.

The infant component, Massage +, begins with 30 s of auditory stimuli (infant directed talk) followed by head-to-foot massage for 10 min, and 5 min of vestibular stimulation (horizontal rocking). The auditory stimuli continue throughout the 15 min intervention. Visual (eye-to-eye) stimuli are offered throughout when the infant becomes alert [56]. Massage + is offered with responsiveness to infant behavioral cues, e.g. pausing for disengagement cues.

### Outcome measure: mother-infant responsivity

Mother-infant responsivity during interaction was measured by the Dyadic Mutuality Code (DMC) during a 5-min play session [57, 58]. The DMC comprises six items: maternal pauses and maternal sensitivity in responsiveness to the infant (parent constructs), infant clarity of cues (infant construct) and mutual attention, positive affect, mutual turn-taking (dyadic constructs) [59]. Each subscale receives a dichotomous global rating of 1 for absent (no occurrence or negative) and 2 for present (occurrence, positive). The total possible score ranges from 6 to 12, with 6–8 categorized as low responsivity, 9 -10 as "moderate responsivity, and 11–12 points as "high responsivity" [57, 58, 60]. In Censullo’s previous work, responsivity was divided into two categories, low responsivity versus medium and high responsivity, with low responsivity regarded as indicating a potential problem in the mother-infant relationship. Higher dyadic responsivity is associated with improved infant development [49, 60].

The session was video recorded and coded by two experts, one who was blinded to group assignment. Both inter- and intra-observer agreement were maintained at a 95% confidence interval in the intra-class correlation coefficient. Using Cohen’s Kappa statistic (k), the intra and inter-rater reliability was found to be 0.90 (95% CI, 0.825 to 0.974, *p* < 0.005) and 0.78 (95% CI, 0.660 to. 899,* p* < 0.005) which are interpreted as very good and good agreement respectively [61]. Internal consistency of the DMC was evaluated through Cronbach coefficient alpha and it was 0.73 (α = 0.95).This is considered acceptable [62].

### Procedures

After the study was approved by the College of Medicine Research and Ethics Committee (COMREC), the study was explained to eligible mothers. Those who agreed to participate signed written informed consent. Mothers under 18 years of age gave written and signed assent and their legal guardians gave signed informed permission (consent). Informed consent was obtained from a parent and/or legal guardian for study participation for infants. Mother-preterm infant dyads were enrolled when the infant was clinically stable and was transferred to the KMC unit. Infants participated in the study until they reached 6-weeks CA. For the H-HOPE + KMC cohort, the research assistants provided the participatory guidance sessions for the mothers using individualized and cohort instructions with hands-on practice and return demonstrations. They also supported the mothers in administering Massage + during hospitalization.

For infants who were born between 29 and 31 weeks gestation, Massage + was provided from 32 weeks post-menstrual age (PMA). For infants born at 32 to 34 weeks gestation, Massage + began immediately after recruitment. Mothers administered Massage + twice a day for 15 min from the time the infant reached 32 weeks PMA and weighed at least 1000 g. After hospital discharge, mothers were told to continue to provide the Massage + at home twice daily until the infant reached one month CA.

Two aspects of fidelity to H-HOPE were documented; the number of times mothers provided Massage + and the fidelity of Massage + procedure when given by mothers. To determine whether mothers were providing Massage + for their infants, mothers kept a record of the number of times they gave their infant Massage + both in the hospital and at home. Mothers were asked to continue to keep a record on the log they were given after they took their infant home through 4 weeks corrected age. Mothers recorded when they completed the intervention on the Massage + log. Of the 60 mothers in H-HOPE, only 4 mothers recorded that on a few days they had provided Massage + only once a day. The mothers in the H-HOPE cohort recorded a total of 15,428 ATVV sessions from 32 weeks GA to 1-month CA. The minimum number of sessions provided was 90 and maximum was 125 sessions. The mean number of massages given per infant was 111 (SD = 8.9). These records documented that mothers provided Massage + regularly and most of them adhered to the recommended frequency of at least twice daily. Fidelity of the mother’s performance on Massage + administration was confirmed during follow-up visits where the research team observed the mother administer Massage + .

Mother-infant dyads were videotaped during a 5-min play session at 6-weeks CA. Mother-infant responsivity was rated via review of the video recording.

#### Data analysis

Before we could test whether the primary outcome, mother-infant responsivity, differed for the control and intervention cohorts, we first assessed whether we could combine the results of the two KMC only cohorts. Mother-infant responsivity for the two KMC only cohorts did not differ (*t* = -0.758, *p* = 0.451), therefore, the data were combined into a single KMC only cohort.

We next examined maternal and infant sample characteristics using descriptive statistics and *t* tests to identify significant differences at enrolment between the two study cohorts. Finally, we examined mother-infant responsivity. We compared the H-HOPE + KMC and KMC only cohorts for both the mean responsivity scores and the proportion of mother-infant dyads with low, moderate, and high responsivity using *t*-tests and chi-square test of significance.

Following the recommendation of Censullo, we then combined moderate and high responsivity into a single category and conducted logistic regression comparing dyads with moderate-to-high responsivity versus low responsivity. We examined the bivariate relationship of study condition and each of the mother and infant characteristics with mother-infant responsivity. Study condition and mother and infant characteristics that were significantly related to responsivity were fit into a multivariate logistic regression model. Throughout the analysis, the level of significance was set at *p* =  < 0.05.

## Results

### Sample characteristics

Maternal and infant characteristics for the total sample and the two study conditions (KMC only and H-HOPE + KMC) are in Table 1. The mean maternal age was 24.7 (*SD* = 6.9). The KMC only cohort had 54% (*n* = 27) of primiparous mothers compared to 46% (*n* = 23) who were in the H-HOPE + KMC cohort. The sample included 48% male and 52% female infants. Most of the infants (91.6%, *n* = 109) were born vaginally and at 33–34 weeks gestational age (72.3%, *n* = 86), and mean birth weight was 1707.9 g. None of these differences were statistically significant (Table 1).

**Table 1:** Maternal and infant characteristics for KMC Only and H-HOPE + KMC groups

	**Total (*n* = 119)**	**KMC only (*n* = 59)**	**H-HOPE + KMC (*n* = 60)**	**Chi-square**	***p* value 0.05**
**Variable**	***n* (%)**	***n* (%)**	***n* (%)**		
**Maternal characteristics**					
**Age, years**				.25	0.884
19 and below	41 (34.5)	21 (35.6)	20 (33.3)		
20–29	45 (37.8)	21 (35.6)	24 (40.0)		
30 and above	33 (27.7)	17 (28.8)	16 (26.7)		
**Marital status**				.251	0.616
Married	103 (86.6)	52 (88.1)	51 (85.0)		
Single	16 (13.4)	7 (11.9)	9 (15.0)		
**Parity**				.404	0.525
Primipara	49 (41.2)	26 (44.1)	23 (38.3)		
Multipara	70 (58.8)	33 (55.9)	37 (61.7)		
**ANC initiation, weeks**				17.851	< 0.001^*^
6–12	50 (42.0)	36 (61.0)	14 (23.3)		
13–27	60 (50.4)	19 (32.2)	41 (68.3)		
28–40	9 (7.6)	4 (6.7)	5 (8.3)		
**Antenatal visits**				8.06	< 0.01^*^
3 or less	87 (73.1)	50 (84.7)	37 (61.7)		
4 or more	32 (26.9)	9 (15.3)	23 (38.3)		
**Infant characteristics**					
**Gestational age, weeks**				1.488	0.475
29–30	4 (3.4)	1 (1.7)	3 (5.0)		
31–32	29 (24.4)	13 (10.9)	16 (26.7)		
33–34	86 (72.3)	45 (37.8)	41 (68.3)		
**Mode of birth**				.001	0.978
Vaginal	109 (91.6)	54 (91.5)	55 (91.7)		
Caesarean Section	10 (8.4)	5 (8.5)	5 (8.3)		
**Sex**				.408	0.523
Male	57 (47.9)	30 (50.8)	27 (45.0)		
Female	62 (52.1)	29 (49.1)	33 (55.0)		
**Birthweight, grams**				1.65	0.438
1500 or less	39 (32.8)	22 (37.3)	17 (28.3)		
1501–1999	55 (46.2)	27 (45.8)	28 (46.7)		
2000–2499	25 (21.0)	10 (16.9)	15 (25.0)		

^*^ = level of significance set at ≤ 0.05, *KMC* Kangaroo Mother Care, *KMC* + *H HOPE* Kangaroo Mother Care plus Hospital to Home transition Optimizing Preterm Infant Environment intervention, *ANC* Antenatal Care

To determine whether the cohort design resulted in comparable cohorts, we examined differences in maternal and infant characteristics at enrollment. Only three maternal characteristics were significantly different between the KMC only cohort and the H-HOPE + KMC cohort (Table 1). Mothers in the H-HOPE + KMC cohort were more likely to have two or more children and to have attended 4 or more ANC visits. There were significantly more mothers in the KMC only cohort who initiated antenatal care (ANC) in the first trimester than those in the H-HOPE + KMC cohort. There was only one significant difference in infant baseline characteristics between the 2 cohorts. More infants in the KMC only cohort had a respiratory rate which was higher than normal on enrollment (more than 60 breaths per minute). Thus, maternal and infant characteristics the H-HOPE + KMC and KMC only cohorts were adequately similar.

### Mother-preterm infant responsivity at 6-weeks CA

**Fig. 2:**
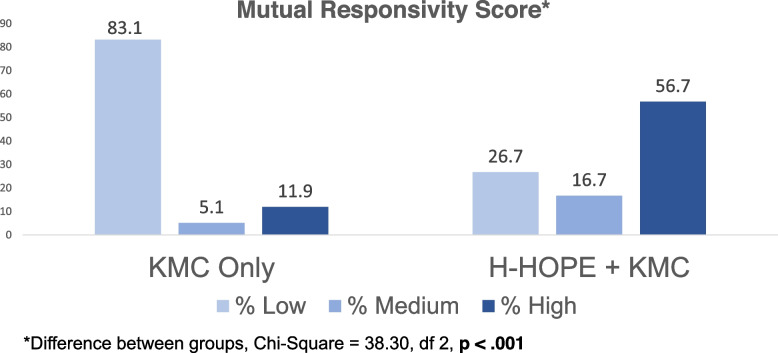
Mother-infant responsivity at 6-weeks CA for H-HOPE + KMC and KMC only dyads

Mothers and their preterm infants in the H-HOPE + KMC cohort exhibited significantly higher overall responsivity at 6-weeks CA compared with the KMC only cohort. The differences in the means of the H-HOPE + KMC and the KMC only cohorts (x̄ = 9.6 vs 7.4 respectively) reached significance (*t* = 7.683, *p* =  < 0.001). We then examined the 3 levels of responsivity. Far fewer dyads in the H-HOPE + KMC versus KMC only cohort exhibited low responsivity (26.7% vs 83.1%). More than half (56.7%) of the dyads assigned to the H-HOPE + KMC cohort exhibited high responsivity, compared to 11.9% in the KMC only cohort (Fig. 2).

### Multivariate predictors of responsivity

We then conducted a multivariate logistic regression examining factors that predicted high vs. low mother-infant responsivity (Table 2). No maternal factors which included age, marital status, parity, ANC initiation and number of ANC visits were related to responsivity. The bivariate analysis had four variables only that were associated with responsivity and these were all included in the multivariate analysis. However, in the multivariate analysis, only study condition and type of delivery remained significant predictors of responsivity. Infants born by vaginal delivery were 5 times more likely to exhibit positive mother-infant responsivity than those born by Caesarean Section. However, it is important to note that only 10 mothers, five in each cohort, had a Caesarean delivery. Infants in the H-HOPE + KMC intervention cohort were 11 times more likely to exhibit positive mother-infant interaction responsivity than those in the KMC cohort only. The impact of being in the H-HOPE + KMC intervention on responsivity increased when the other significant predictor was controlled. We also calculated the effect size for being in the H-HOPE + KMC cohort, (1.44), indicating an effect greater than one standard deviation.

**Table 2:** Logistic regression: Positive mother-infant responsivity for KMC + H-HOPE dyads vs. KMC only dyads

Variable	Univariate		Multivariate	
	**Odds Ratio (95% CI)**^**a**^	***p* value**	**Adjusted Odds ratio (95% CI)ª**	***p* value**
Type of delivery (Vaginal)	2.97 (.73, 12.08)	< .001^*^	5.44 (0.1, 30.96)	.02^*^
Birthweight	3.3 (1.2, 9.2)	.02^*^	1.11 (0.34, 3.65)	.86
Birthweight regained by 10 days	1.3 (0.6, 2.8)	.05^*^	1.32 (0.27, 1.74)	.58
H-HOPE + KMC	8.03 (.04, 20)	< .001^*^	11.51 (4.56, 29.04)	< .001^*^

^a^*CI* Confidence Interval

## Discussion

In this study, at 6-weeks CA mother-preterm infant dyads who received both H-HOPE and KMC had substantially higher responsivity than those who received KMC only. This finding is congruent with the randomized controlled trial in the US where the H-HOPE intervention facilitated establishment of higher responsivity during play as well as more maternal social-emotional growth fostering behaviors and greater infant clarity of cues during feeding [46]. This is the only study that can be compared directly with the Malawi study because both studied preterm infants using the same measure of responsivity (DMC) and the same assessment time (6-weeks CA).

Notably, the effect size for our study in Malawi was very large (1.44), much larger that the effect size of 0.30 in the US study. The biggest difference in the responsivity between the two studies was the much higher proportion of KMC only dyads who had low responsivity scores. Reducing the number of mother-infant dyads with low responsivity at 6-weeks CA has high clinical relevance, as the developer of the DMC identified that low responsivity was an indicator for future mother-infant interaction problems that would warrant follow-up [57, 58].

The larger H-HOPE effect size in the Malawi study likely reflects major differences in the context of care and that KMC is Malawi’s standard of care. These two interventions together may enhance the mother-infant relationship more than H-HOPE alone. KMC provides mothers with constant physical contact with their infants, promoting closeness. H-HOPE teaches mothers to read, interpret, and respond to their infant’s cues and provides opportunities for mother-infant social interaction, including eye-to-eye contact and vocalizations during Massage +.

In Malawi a much larger proportion of dyads had low responsivity compared to the US study. Although providing KMC 24 h a day is good for the premature infant, the lack of engagement with relatives and friends is difficult, stressful, and isolating for the mothers [21]. The stress mothers experience when providing KMC in the hospital may be a major factor that slows the development of mother-infant responsivity. When mother-infant dyads experience H-HOPE this may foster optimal development of mother-infant responsivity.

Vaginal delivery (vs. Caesarean section) was the only other factor that predicted a higher mother-infant responsivity. Possible reasons for this relationship include the reasons for this type of delivery, such as preeclampsia or hemorrhage [63], inadequate pain relief, fatigue and difficulty in moving about, that could compromise mothers’ capability to fully engage with their infants. However, given that only 10 mothers in our sample had a Caesarean Section (five in each study condition), this pattern needs to be examined more closely in future studies.

Although individual level randomization is the gold standard, this design is not possible given that there are groups of mother-infant dyads in the KMC unit. Only 65% mothers returned to the hospital at 6-weeks CA, when responsivity was assessed. Returning to the hospital probably required more time and/or greater expense than a visit to the local clinic where mothers are usually referred. We extended recruitment to obtain a final sample with adequate power, but we cannot rule out the possibility that mothers who returned differed from those who did not in ways that were not measured. Finally, most mothers did not receive a prenatal ultrasound, so gestational age in the medical record was estimated by recall of last menstrual period (LMP) and fundal height assessment. However, gestational assessment by LMP and fundal height is not very reliable [64–66]. To mitigate this limitation, when no ultrasound was available, we used the modified Ballard Index [55] to determine a more accurate gestational age.

## Implications

This study demonstrated that H-HOPE can be provided in Malawi and has a high impact on the development of early mother-infant responsivity, fostering positive parent-infant relationships. H-HOPE is complementary to KMC, which is already the standard of care for preterm infants in Malawi. Incorporating H-HOPE along with KMC for mother-preterm infant dyads as the standard of care can support early parent-infant responsivity, infant feeding, and development. Changing clinical practice to include H-HOPE has the potential to dramatically reduce the number of mother-infant dyads with low responsivity. Improving early mother-preterm infant responsivity can avert the need for later follow-up, which is relatively unavailable in Malawi as well as many other low-resource countries.

Introducing H-HOPE as part of the standard of care for preterm infants would offer substantial benefits for preterm infants and their mothers, supporting early infant development and strengthening the mother-infant relationship that is crucial for infants to thrive. However, in this study, H-HOPE was offered by a nurse research assistant. Having nurses provide H-HOPE is probably not feasible for widespread introduction of H-HOPE due to the nursing shortage in Malawi. A patient attendant is always present in the KMC unit, and the patient attendants expressed high interest in the H-HOPE program. If patient attendants can be trained to provide H-HOPE with fidelity, introducing H-HOPE would be highly cost-effective. H-HOPE offers a critical early behavioral intervention to support preterm infants and their mothers. Offering H-HOPE as the standard of care complementary to KMC can contribute to the achievement of the WHO’s Sustainable Development Goal Number 3, to ensure optimal health and promote mother and infant well-being [67].

## Definitions

Corrected Age (CA): chronological age of infant reduced by the number of weeks born before 40 weeks of gestation

## Data Availability

The datasets analyzed during the current study are not publicly available but will be made available from the corresponding author on reasonable request.

## Abbreviations

ANC: Antenatal care
ATVV: Auditory, Tactile, Visual and Vestibular stimulation
DMC: Dyadic Mutuality Code
H-HOPE: Hospital to Home: Optimizing the Preterm Infant Environment
HIV: Human Immunodeficiency Virus
KMC: Kangaroo Mother Care
NICU: Neonatal Intensive Care Unit
US: United States of America
WHO: World Health Organization
